# Thyroid function derangement and childhood obesity: an Italian experience

**DOI:** 10.1186/1472-6823-10-8

**Published:** 2010-05-04

**Authors:** Anna Grandone, Nicola Santoro, Filomena Coppola, Paolo Calabrò, Laura Perrone, Emanuele Miraglia del Giudice

**Affiliations:** 1 Department of Pediatrics "F. Fede", Seconda Università degli Studi di Napoli, Via Luigi De Crecchio 2, 80138, Napoli, Italy; 2 Division of Cardiology, Seconda Università degli Studi di Napoli -A.O.Monaldi, Via L.Bianchi, 80131, Napoli, Italy

## Abstract

**Background:**

In recent years, there has been an increasing attention to thyroid function in paediatric obese patients. In the present study we aimed 1) to determine the prevalence of abnormally elevated thyroid-stimulating hormone (TSH) levels in Italian obese children and adolescents 2) to investigate whether hyperthyrotropinemia in obese children cardiovascular and metabolic risk factors 3) to verify if TSH elevation is reversible after weight loss.

**Methods:**

We examined 938 obese children and adolescents (450 females). Anthropometric, metabolic and hormonal variables were determined at baseline and, in a subgroup of children with hyperthyrotropinemia, after a six month weight loss program.

**Results:**

Hyperthyrotropinemia (TSH ≥4.2 μUI/ml) was diagnosed in 120 patients (12,8%). Body mass index (BMI) z-score (p = 0.02) and free T3 (fT3) levels (p = 0.03) were higher in patients with elevated TSH compared to the group with normal TSH. There were not significant differences in other metabolic parameters between the two groups.

A positive correlation between baseline TSH and BMI z-score (p = 0.0045) and between Ft3 and BMI z-score (p = 0.0034) was observed, while there was no correlation between TSH and lipids. Twenty-three patients among those with hyperthyrotropinemia who participated to weight reduction intervention (64 patients), presented substantial weight loss and concomitantly a significant decrease in TSH and in fT3.

**Conclusions:**

These results suggest that: (1) a moderate elevation of TSH concentrations, is frequently found in obese children; (2) in obese children increase of TSH is not associated to metabolic risk factors, (3) hyperthyrotropinemia is reversible after weight loss and these data suggest that it should not be treated.

## Background

Obesity is considered a worldwide health problem and its prevalence is increasing steadily and dramatically all over the world [1]. Paediatricians are often involved in the initial evaluation of paediatric obesity and its numerous co-morbidities. Obese individuals are, in fact, at high risk of developing dyslipidemia, hypertension and impaired glucose tolerance, with the consequent increase of their risk of metabolic and cardiovascular diseases [2].

In recent years, there has been an increasing attention to thyroid function in paediatric obese patients [3]. Hypothyroidism has often been thought to be the cause of obesity, and thyroid function tests are still now one of the most commonly performed laboratory analysis in this population. It has been demonstrated by several studies that obese children show higher thyroid-stimulating hormone (TSH) levels than normal weight subjects [3-7], with a higher prevalence of TSH elevation. Isolated hyperthyrotropinemia is a condition characterized by a serum TSH above the statistically defined upper limit of the reference range with normal or slightly high serum free T4 (fT4) and free T3 (fT3) concentration [8]. Whether or not increased TSH levels affect the metabolic and cardiovascular profile in obese children and adolescents remains unclear, as well as TSH decrease after weight loss [6,7]. Thereby, there still is considerable disagreement regarding treatment.

In order to achieve a better understanding of this issue we aimed 1) to determine the prevalence of hyperthyrotropinemia in obese children and adolescents 2) to investigate whether hyperthyrotropinemia in childhood obesity is related to dyslipidemia and/or other metabolic complications 3) to verify whether hyperthyrotropinemia may reverse after weight loss program. Our series is the largest published till now.

## Methods

We examined 1010 children and adolescents, referred to our ward (Childhood Obesity Service, Department of Paediatrics, Second University of Naples) for obesity between 1999 and 2008. Subjects with known presence of diabetes or using medications altering blood pressure, glucose or lipid metabolism, with goitre or known thyroid disease were excluded (1 subject with type 2 diabetes, 9 with autoimmune thyroiditis). The ethical committee of the Second University of Study of Naples approved the study. Informed consent was obtained by parents and, where appropriate, by children.

Of the 1010 subjects enrolled, 485 were girls. Obesity was defined according to the body mass index (BMI) 95^th ^percentile for age and sex using the definition of the International Task Force for Obesity in Childhood and the charts for Italian population [9]. Obesity degree was evaluated using the z-score BMI, calculated with the LMS method [10].

Anthropometric measures were assessed at the time of the first visit and in a subgroup of patients with TSH elevation after 6 months.

Waist circumference was measured by the same operator to the nearest centimetre with a flexible steel tape measure while the subjects were standing, after gently exhaling, as the minimal circumference measurable on the horizontal plane between the lowest portion of the rib cage and iliac crest. The intra-operator coefficient of variation was 1,3%.

Standard deviation (SD) scores for waist circumference have been calculated using normative values for Italian population [11].

Systolic blood pressure (SBP) and diastolic blood pressure (DBP) were measured three times while the subjects were seated, and the two last measurements were averaged for the analysis and we calculated SD according to normative values [12].

The pubertal stage was assessed using Tanner criteria [13].

After an overnight fasting, blood sample were obtained for triglycerides, high density lipoprotein (HDL) cholesterol, insulin, serum glucose, thyroid hormones (TSH, fT3, fT4) and anti-tyreoglobulin (Tg-Ab) and anti-peroxidase anti-bodies (TPO-Ab).

Serum fasting glucose levels were measured with glucose oxidase method. Triglycerides and HDL cholesterol were measured by an Olympus AU 560 apparatus using an enzymatic colorimetric method.

Immunoreactive insulin was assayed by IMX (Abbott Diagnostics, Santa Clara, CA). The mean intra- and inter-assay coefficients of variations were 4.7% and 7.2%, respectively. The degree of insulin resistance was determined using a homeostasis model assessment (HOMA), [insulin (mU/L) × glucose level(mmol/L)/22.5] [14].

Thyroid hormones (TSH, fT3, and fT4) and TPO-Ab and Tg-Ab were determined by high-specific solid-phase technique-chemiluminescenceimmunoassays (Perkinelmer, Turku, Finland).

In a subgroup of 160 children we measured leptin plasma levels using double-antibody radioimmunoassay (Human Leptin RIA KIT, Linco-Research, St. Charles, MO). The sensitivity cut-off for the leptin assay was 0.5 ng/ml; the intra and inter-assay variation coefficients were 10.8% and 7%, respectively.

TPO-Ab and Tg-Ab levels higher than 60 UI/ml were considered positive, and autoimmune thyroiditis was diagnosed.

Isolated hyperthyrotropinemia was diagnosed when TSH was higher than 4.2 μUI/ml (97,5^th ^for our assay), with normal fT3 and fT4 and no signs or symptoms of hypothyroidism.

All patients were submitted to a weight loss program. They consumed a nutritionally balanced (50% carbohydrate, 30% fat and 20% protein) self-selected diet of common foods (80% of the recommended dietary energy allowances for age and sex). All subjects underwent a lifestyle modification program. They followed a program based on physical exercise, and behavioral therapy, including individual psychological care of the child and his or her family. Thyroid hormones and lipids were reassessed after 6 months of the weight reduction program in patients with elevated TSH levels. Weight reduction was considered as at least a 0.5 decrease in BMI z-score [15].

All values are expressed as mean ± SD. Skewness and kurtosis tests were used to evaluate the normal distribution of the variables. The significance of changes in discrete variables was analyzed by the X^2 ^test.

Mean differences were analyzed with one-way ANOVA for variables with a normal distribution, and with the Kruskal-walis test for variables with non Gaussian distribution. Obese children with isolated hyperthyrotropinemia, were compared to the obese children with normal TSH concentrations with respect to peripheral hormones, lipids, HOMA-index, blood pressure and anthropometric measures, and in a subgroup to leptin.

In the patients with hyperthyrotropinemia independent samples t-test for normal distribution and Mann-Whitney test for parameters with non-Gaussian distribution were used to compare the changes in the outcome variables between the group with decrease in BMI z-score >0.5 point, the one with decrease in BMI z-score between 0.2 and 0.5 points and the group with no changes in BMI z-score.

Linear regression model has been performed to investigate the correlation between TSH and BMI z-score, waist, lipids, blood pressure and HOMA index (and leptin in the subgroup in which it was tested).

Multiple linear regression analyses with lipids or blood pressure as dependent variables and age, gender, degree of overweight (BMI z-score) pubertal stage, HOMA index, and thyroid hormones as independent variables were performed.

Gender and pubertal stage were used as classified variables. A *P *< 0.05 was considered statistically significant. Stat-Graph 3.0 software for Windows was used for the statistical analysis.

## Results

The anthropometric and biochemical characteristics of patients are shown in Table 1. Overt hypothyroidism was diagnosed in 12 (1,2%) patients, all with positive thyroidal auto-antibodies; these patients were excluded from the analysis.

**Table 1:** Anthropometric, clinical and biochemical characteristics at baseline of 938 obese children involved in the study.

	Mean ± SD	Range
N	938	

Prepubertal (%)	572 (61%)	

Age (yr)	10.3 ± 2.6	4.5-15.9

Gender	48% girls	

BMI z-score	2.9 ± 0.7	1.8-5.8

Waist SD	3.7 ± 2.3	0-11

SBP SD	0.7 ± 1.1	-1.5-4.6

DBP SD	0.2 ± 0.7	-1.3-2.7

TSH (micrUI/ml)	2.6 ± 1.2	0.2-8.5

fT3 (pg/ml)	3.8 ± 0.8	1.1-7.1

fT4 (pg/ml)	10.3 ± 1.6	6.2-15.9

HDL (mg/dl)	45 ± 9.5	28-82

Triglycerides (mg/dl)	91.2 ± 44	50-198

HOMA	7.3 ± 5.4	1.1-18

Other 60 subjects had positive auto-antibodies, 8 of which with subclinical hypothyroidism, and were all excluded from the analysis; in total 72 patients (7%) had autoimmune thyroiditis confirmed by ultrasonography.

Isolated hypethyrotropinemia (TSH≥4.2 μU/l) was diagnosed in 120 patients (62 girls) of the remaining 938 patients enrolled (12,8%), 70% were prepubertal. There were no significant differences by sex and pubertal stage (p = 0.2 and p = 0.4 respectively) compared to those with normal TSH levels.

Obese children with and without hyperthyrotropinemia had fT3 and fT4 concentrations within the normal range and did not differ significantly in their fT4, lipid concentrations, HOMA values and blood pressure compared to those with TSH in the normal range (Table 2). All analysis were adjusted for age, sex and pubertal stage.

**Table 2:** Anthropometric, clinical and biochemical characteristics of obese patients with hyperthyrotropinemia compared to those with normal TSH.

	TSH ≥4.2 micrUI/ml	TSH <4.2 micrUI/ml	P value
N	120 (12.7%)	818	

Age (yr)	9.7 ± 2.7	10.4 ± 2.6	0.0007

Gender	52% girls	47% girls	>0.05

BMI z-score	3.1 ± 1	2.8 ± 0.7	0.02

Waist SD	3.4 ± 2.2	3.5 ± 2.2	>0.05

SBP SD	0.9 ± 1.2	0.7 ± 1.1	>0.05

DBP SD	0.17 ± 0.8	0.16 ± 0.7	>0.05

TSH (μUI/ml)	5.1 ± 1	2.3 ± 0.8	<0.00001

fT3 (pg/ml)	4 ± 0.9	3.8 ± 0.8	0.03

fT4 (pg/ml)	10.3 ± 1.8	10 ± 1.7	>0.05

HDL (mg/dl)	45.5 ± 9	43.7 ± 9	>0.05

Triglycerides (mg/dl)	94 ± 44	87 ± 38	>0.05

HOMA	7.7 ± 4.6	7.1 ± 5	>0.05

BMI z-score (p:0,02) and fT3 levels were significantly higher (p:0.03) in patients with elevated TSH, while their age was significantly lower (p:0,0007).

In all the 938 patients enrolled a positive correlation between baseline TSH and BMI z-score (p = 0.0045) and between Ft3 and BMI z-score (p = 0.0034), adjusted for age, gender and pubertal stage, was observed.

In a multiple regression analysis adjusted for age, gender, pubertal age, degree of overweight and HOMA, there was no significant correlation between TSH and HDL (p:0,6), triglycerides (p:0,5), blood pressure (p:0,5) as well as between fT3 and HDL (p:0,5), triglycerides (p:0,6), blood pressure (p:0,5) as well as between fT4 and all the mentioned parameters.

In a subgroup (160 patients), randomly selected, we measured plasma leptin. Among this subgroup 22 patients had isolated TSH elevation and their leptin levels were not different from those with normal TSH, adjusting for BMI z-score. Moreover in all 160 patients we did not find any correlation between leptin and TSH, also adjusting for gender, pubertal stage and degree of overweight (data not shown).

A total of 64 patients with elevated TSH levels completed (53%) the weight reduction intervention. No difference in the baseline characteristics were found between patients who dropped out and those who did not. Among these 64 patients, 23 patients presented a substantial weight loss, defined as a decrease at least in 0.5 points of their BMI z-score [15]. Twenty-one patients presented a decrease in BMI z-score between 0.2 and 0.5 points, while the other 20 did not loose weight.

Compared to their baseline parameters, patients who lost substantially weight presented a statistically significant decrease in TSH and in fT3 and improvement in lipid profile; such improvement did not significantly correlate to thyroid hormones after adjusting for changes in BMI (Table 3).

**Table 3:** Degree of overweight, thyroid hormones and lipids concentrations in obese children with hyperthyrotropinemia before and after weight loss program.

	Decrease in BMI-SDS >0.5			Decrease in BMI-SDS 0.2-0.5			No weight loss		
**N**	**23**			**21**			**20**		

**Gender**	**61% girls**			**52% girls**			**45% girls**		

	**Baseline**	**Six months later**	**p**	**Baseline**	**Six months later**	**p**	**Baseline**	**Six months later**	**p**

BMI z-score	2.9 ± 1.3	2.0 ± 1	0.007	2.8 ± 0.5	2.5 ± 0.6	0.3	2.7 ± 0.5	2.7 ± 0.6	0.4

TSH (μUI/ml)	5.1 ± 1	3.6 ± 1.1	0.0001	5.2 ± 0.9	4.6 ± 2.6	0.3	5.27 ± 1.3	4.6 ± 2.6	0,4

fT3 (pg/ml)	3.9 ± 1	3.4 ± 0.5	0.03	3.7 ± 0.7	4 ± 0.2	0.1	3.8 ± 0.7	4.4 ± 0.2	0.09

fT4 (pg/ml)	9.8 ± 2.5	8.8 ± 2.9	0.1	9.8 ± 2.5	10.2 ± 1.8	0.1	10 ± 2	10.3 ± 1.6	0.8

HDL (mg/dl)	40 ± 23	45 ± 14	0.6	37 ± 21	39 ± 14	0.8	37 ± 15	38 ± 13	0.9

Triglycerides (mg/dl)	101 ± 39	90 ± 40	0.6	103 ± 85	98 ± 34	0.9	108 ± 61	98 ± 65	0.8

Patients without weight loss, or with a weight loss less than 0.5 points in their BMI z-score, on the contrary, did not show any difference in TSH and in fT3 levels compared to the values before the weight loss program (Table 3).

## Discussion

The relationship between obesity and thyroid dysfunction is a topic of great interest, as a large number of obese children now seeking medical care.

Isolated TSH elevation has been demonstrated to be quite frequent in obese children, but it is not clear if such condition is associated to increased cardiovascular risk factors, if it reverses after weight loss, and, therefore, there is general disagreement regarding whether and when to start treatment with L-thyroxin [6,7,16].

We believe our series is the larger than any other published report.

In agreement with other studies, isolated hyperthyrotropinemia is quite common in our sample of obese children (12,8%), [3,5-7,17]; a limitation of our study is lacking of a control group, anyway prevalence of isolated hyperthyrotropinemia in our sample is higher compared to the prevalence observed in normal weight subjects (2%), even if epidemiological data on lean children and adolescents are scarce [18-20].

Thyroiditis appears to occur frequently in our sample of obese subjects (7%) compared to the reported prevalence of 1.2% in normal weight children [21]. This condition, therefore, should always be excluded when obese patients show elevated TSH levels; in the present study most part of obese subjects with hyperthyrotropinemia (120 out of 128, i.e. 93.7%) did not have thyroiditis.

The reason of the increase of TSH in obese subjects, both adults and children, is not clear.

Patients with higher levels of TSH had a lower mean age, but the trend of TSH to decrease with age is well known and described [20]. Furthermore, this trend does not affect statistical results as every analysis has been adjusted for age.

We found a direct correlation between TSH and BMI-z-score and between fT3 and BMI-z-score; this relationship could suggest an association with leptin, which is regulated by body adiposity [22]. Furthermore, there is a synchronicity between the secretion of leptin and TSH [23]. A recent report demonstrated that TSH is related both to BMI and to leptin in obese and anorexic patients [24]. However a previous study had shown no correlation between TSH and leptin [25]. We failed to demonstrate an association between leptin and TSH, probably due the homogeneity of the weight status of our patients (we studied only obese subjects). Furthermore, considering that TSH production is regulated by several transmitters and hormones which regulates also body weight and satiation, such as neuropeptide Y, alpha-melanocyte-stimulating hormone and leptin itself [26,27], a mechanism of regulation of TSH more complicated than a simple linear association among TSH and leptin levels, could be inferred to explain this lack of association. For example a tissue-specific modulation of deiodinases at pituitary level might be implicated in the effect of leptin on thyroid function. Studies in animal models show that leptin administration can decrease D2 deiodinase activity in pituitary tissue, thus modifying the feedback of T3 on TSH secretion [28].

In some studies subclinical hypothyroidism has been shown to worsen metabolic profile, causing dyslipidemia or heart dysfunction [29,30]. However there is no agreement on this point and many studies are on adult patients.

Obese children are at increased metabolic risk, because they can present insulin resistance, hypertension or dyslipidemia [2].

We show that obese children with isolated elevation of TSH do not have an increase in their metabolic risk factors and demonstrate no significant relationship between TSH levels and lipids, blood pressure and insulin resistance.

These findings are in agreement with some other studies in adults and children [5,31].

After substantial weight reduction, TSH and fT3 lower significantly and normalize, in accordance with a previous study by Reinehr and in contrast with a recent study by Shalitin et al [5,7]. Slight reduction in weight, that is a decrease in BMI z-score of less than 0.5 points, is not associated to TSH normalization, suggesting that a weight reduction able to modify insulin resistance and cardiovascular risk factor is needed to influence thyroid hormone metabolism.

Obese patients have with hyperthyrotropinemia have fT3 levels significantly higher compared to obese children with normal TSH levels. This finding confirms the theory that the negative feedback between TSH and the peripheral hormones is decreased. Furthermore, fT3 levels diminish after weight loss. These findings can suggest another observation about pathogenesis. Thyroid hormones, especially fT3, regulate the resting energy expenditure (REE). In obese patients the moderate increase in fT3 and TSH lead to an increase of energy expenditure. As a consequence of this, the availability of accumulated energy for conversion into fat is diminished [32]. The alterations of thyroid hormones in obesity have been suggested to be an adaptation process.

If this hypothesis is true, the possibility exists that, as weight loss is associated with a decrease of TSH and fT3, the resulting decrease in REE may contribute towards the difficulties of obese children in maintaining weight loss [8].

## Conclusions

In summary, a moderate elevation of TSH concentration is frequently found in obese children and is not associated to increased metabolic risk.

This condition seems rather a consequence than a cause of obesity since weight loss leads to a normalization of elevated thyroid hormone levels.

There is diffuse disagreement about the management of hyperthyrotropinemia both in adults and children. In particular we lack of large studies that demonstrate advantages to treat children showing TSH between 5 and 10 μU/ml [33].

Considering our results we suggest that obese children with this condition should not be treated under the "diagnosis" of subclinical hypothyroidism because TSH alteration is more a consequence than a cause of obesity.

## Abbreviations

(Tg-Ab): anti-tyreoglobulin anti-bodies; (TPO-Ab): anti-peroxidase anti-bodies; BMI: (body mass index); DPB: (diastolic blood pressure); (fT3): free T3; (fT4): free T4; (HDL): high density lipoprotein; HOMA: (homeostasis model assessment); (REE): resting energy expenditure; SBP: (systolic blood pressure); SD: (standard deviation); (TSH): thyroid-stimulating hormone.

## Competing interests

The authors declare that they have no competing interests.

## Auhors' contributions

AG conceived of the study, participated in the design of the study and performed the statistical analysis. NS participated in the development of the protocol and analytic framework for the study, and contributed to the writing of the manuscript. FC participated in the development of the protocol, recruited patients and was responsible of clinical assessment of patients. PC was responsible for cardiovascular and leptin assessment. LP and EMDG supervised the design and execution of the study, performed the final data analyses, and contributed to the writing of the manuscript. All authors read and approved the final manuscript.

## Pre-publication history

The pre-publication history for this paper can be accessed here:

http://www.biomedcentral.com/1472-6823/10/8/prepub

## References

[B1] Sokol RJ The chronic disease of childhood obesity: the sleeping giant has awakened J Pediatr 2000 136 711 13 10.1067/mpd.2000.107787 10839864

[B2] Nathan BM Moran A Metabolic complications of obesity in childhood and adolescence: more than just diabetes Curr Opin Endocrinol Diabetes Obes 2008 15 21 29 18185059 10.1097/MED.0b013e3282f43d19

[B3] Stichel H l'Allemand D Gruters A Thyroid function and obesity in children and adolescents Horm Res 2000 54 14 19 10.1159/000063431 11182630

[B4] Bhowmick SK Dasari G Levens KL Rettig KR The prevalence of elevated serum thyroid-stimulating hormone in childhood/adolescent obesity and of autoimmune thyroid disease in a subgroup J Natl Assoc 2007 99 773 776 PMC2574343 17668643

[B5] Reinehr T de Sousa S Andler W Hyperthyrotropinemia In obese children is reversible after weight loss and is not related to lipids J Clin Endocrinol Metab 2006 91 3088 3091 10.1210/jc.2006-0095 16684827

[B6] Reinher T Andler W Thyroid hormones before and after weight loss in obesity Arch Dis Child 2002 87 320 323 10.1136/adc.87.4.320 12244007 PMC1763034

[B7] Shalitin S Yackobovitch-Gavan M Philip M Prevalence of thyroid dysfunction in obese children and adolescents before and after weight reduction and its relation to other metabolic paramenters Horm Res 2009 71 155 161 10.1159/000197872 19188740

[B8] Reinher T Obesity and thyroid function Mol Cell Endocrinol 2009 in press 10.1016/j.mce.2009.06.005 19540303

[B9] Cacciari E Milani S Balsamo A Spada E Bona G Cavallo L Italian crosssectional growth charts for height, weight and BMI (2 to 20 yr) J Endocrinol Invest 2006 29 581 93 16957405 10.1007/BF03344156

[B10] Cole TJ The LMS method for constructing normalized growth standards Eur J Clin Nutr 1990 44 45 60 2354692

[B11] Zannolli R Morgese G Waist percentiles: a simple test for atherogenic disease? Acta Paediatr 1996 85 1368 9 10.1111/j.1651-2227.1996.tb13928.x 8955469

[B12] National High Blood Pressure Education Program Working Group on High Blood Pressure in Children and A The fourth report on the diagnosis, evaluation, and treatment of high blood pressure in children and adolescents Pediatrics 2004 114 555 76 10.1542/peds.114.2.S2.555 15286277

[B13] Tanner JM Whitehouse RH Clinical longitudinal standards for height, weight, height velocity, weight velocity, and stages of puberty Arch Dis Child 1976 51 170 9 10.1136/adc.51.3.170 952550 PMC1545912

[B14] Matthews DR Hosler JP Rudenski AS Naylor BA Treacher DF Turner RC Homeostasis model assessment: insulin resistance and beta-cell function from fasting plasma glucose and insulin concentration in man Diabetologia 1985 28 412 419 10.1007/BF00280883 3899825

[B15] Reinehr T Andler W Changes in the atherogenic risk factor profile according to degree of weight loss Arch Dis Child 2004 89 419 422 10.1136/adc.2003.028803 15102630 PMC1719907

[B16] Eliakim A Barzilai M Walach B Nemet D Should we treat elevated thyroid stimulating hormone levels in obese children and adolescents? Int J Pediatr Obes 2006 1 217 221 10.1080/17477160600805006 17907328

[B17] Reinehr T Andler W Thyroid hormones before and after weight loss in obesity Arch Dis Child 2002 87 320 10.1136/adc.87.4.320 12244007 PMC1763034

[B18] Cetinkaya E Aslan AT Vidinlisan S Ocal G Height improvement by L-thyroxine treatment in subclinical hypothyroidism Pediatr Int 2003 45 534 10.1046/j.1442-200X.2003.01786.x 14521527

[B19] Wu T Flowers JW Tudiver F Wilson JL Punyasavatsut N Subclinical thyroid disorders and cognitive performance among adolescents in the United states BMC Pediatr 2006 6 12 10.1186/1471-2431-6-12 16623938 PMC1459154

[B20] Kapelari K Kirchlechner C Högler W Schweitzer K Virgolini I Moncayo R Pediatric reference intervals for thyroid hormone levels from birth to adulthood: a retrospective study BMC Endocrine Disorders 2008 8 15 10.1186/1472-6823-8-15 19036169 PMC2645400

[B21] Rallison ML Dobyns BM Keating FR Rall JE Tyler FH Occurrence and natural history of chronic lymphocytic thyroiditis in childhood J Pediatr 1975 86 675 10.1016/S0022-3476(75)80350-7 48541

[B22] Ortiga-Carvalho TM Oliveira KJ Soares BA Pazos-Moura CC The role of leptin in the regulation of TSH secretion in the fed state: in vivo and in vitro studies J Endocrinol 2002 174 121 125 10.1677/joe.0.1740121 12098670

[B23] Mantzoros CS Ozata M Negrao AB Suchard MA Ziotopoulou M Caglayan S Synchronicity of frequently sampled thyrotropin (TSH) and leptin concentrations in healthy adults and leptin-deficient subjects: evidence for possible partial TSH regulation by leptin in humans J Clin Endocrinol Metab 2001 86 3284 91 10.1210/jc.86.7.3284 11443202

[B24] Reinehr T Isa A de Sousa G Dieffenbach R Andler W Thyroid hormones and their relation to weight status Horm Res 2008 70 51 57 10.1159/000129678 18493150

[B25] Sreenan S Cairo JF Refetoff S Thyroid dysfunction is not associated with alteration in serum leptin levels Thyroid 1997 7 407 409 10.1089/thy.1997.7.407 9226211

[B26] Mihaly E Fekete C Tatro C Liposits Z Stopa EG Lechan RM Hypophysiotropic thyrotropin-releasing hormone-synthesizing neurons in the human hypothalamus are innervated by neuropeptide Y, agouti-related protein, and alpha-melanocyte-stimulating hormone J clin Endocrinol Metab 2000 85 2596 2603 10.1210/jc.85.7.2596 10902813

[B27] Syed MA Thompson MP Pachucki J Burmeister LA The effect of thyroid hormone on size of fat depots accounts for most of the changes in leptin mRNA and serum levels in the rat Thyroid 1999 9 503 12 10.1089/thy.1999.9.503 10365683

[B28] Araujo RL Andrade BM da Silva ML Ferreira AC Carvalho DP D2 deiodinase activity at pituitary level, which controls the feedback of T3 on TSH Am J Physiol Endocrinol Metab 2009 296 E1157 63 10.1152/ajpendo.90869.2008 19208852

[B29] Rodondi N Aujesky D Vittinghoff E Cornuz J Bauer DC Subclinical hypothyroidism and the risk of coronary heart disease: a meta-analysis Am J Med 2006 119 541 51 10.1016/j.amjmed.2005.09.028 16828622

[B30] Hueston WJ King DE Geesey ME Serum biomarkers for cardiovascular inflammation in subclinical hypothyroidism Clin Endocrinol (Oxf) 2005 63 582 587 10.1111/j.1365-2265.2005.02388.x 16268812

[B31] De Pergola G Ciampolillo A Paolotti S Trerotoli P Giorgino R Free triiodothyronine and thyroid stimulating hormone are directly associated with waist circumference, independently of insulin resi stance, metabolic parameters and blood pressure in overweight and obese women Clin Endocrinol 2007 67 265 269 10.1111/j.1365-2265.2007.02874.x 17547687

[B32] Kiortis DN Durack I Turpin G Effects of a low-calorie diet on resting metabolic rate and serum triiodothyroxine levels in obese children Eur J Pediatr 1999 158 446 450 10.1007/s004310051117 10378389

[B33] Niedziela M Subclinical hypothyroidism: dilemmas in the treatment of children J Endocrinol Invest 2007 30 529 531 17646731 10.1007/BF03346340

